# Limited Heme Oxygenase Contribution to Modulating the Severity of *Salmonella enterica* serovar Typhimurium Infection

**DOI:** 10.3390/antiox11061040

**Published:** 2022-05-24

**Authors:** Valentina P. Sebastián, Daniela Moreno-Tapia, Felipe Melo-González, María P. Hernández-Cáceres, Geraldyne A. Salazar, Catalina Pardo-Roa, Mónica A. Farías, Omar P. Vallejos, Bárbara M. Schultz, Eugenia Morselli, Manuel M. Álvarez-Lobos, Pablo A. González, Alexis M. Kalergis, Susan M. Bueno

**Affiliations:** 1 Millenium Institute on Immunology and Immunotherapy, Departamento de Genética Molecular y Microbiología, Facultad de Ciencias Biológicas, Pontificia Universidad Católica de Chile, Santiago 8331150, Chile; vpsebast@uc.cl (V.P.S.); dpmoreno@uc.cl (D.M.-T.); famelo@bio.puc.cl (F.M.-G.); geraldyne.salazar.t@mail.pucv.cl (G.A.S.); cpardo1@uc.cl (C.P.-R.); mrfarias@uc.cl (M.A.F.); opvallejos@uc.cl (O.P.V.); bmschult@uc.cl (B.M.S.); pagonzalez@bio.puc.cl (P.A.G.); akalergis@bio.puc.cl (A.M.K.); 2 Laboratory of Autophagy and Metabolism, Departamento de Fisiología, Facultad de Ciencias Biológicas, Pontificia Universidad Católica de Chile, Santiago 8331150, Chile; mpherna2@uc.cl (M.P.H.-C.); emorselli@bio.puc.cl (E.M.); 3 Autophagy Research Center, Santiago 8331150, Chile; 4 Instituto de Investigación en Ciencias Odontológicas, Facultad de Odontología, Universidad de Chile, Santiago 8380544, Chile; 5 Departamento de Gastroenterología, Facultad de Medicina, Pontificia Universidad Católica de Chile, Santiago 8331150, Chile; alvarezl@med.puc.cl; 6 Departamento de Endocrinología, Facultad de Medicina, Pontificia Universidad Católica de Chile, Santiago 8331150, Chile

**Keywords:** *S*. Typhimurium, heme oxygenase 1, cobalt protoporphyrin-IX, tin protoporphyrin-IX, autophagosome, autolysosome, autophagy

## Abstract

An important virulence trait of *Salmonella enterica* serovar Typhimurium (*S.* Typhimurium) is the ability to avoid the host immune response, generating systemic and persistent infections. Host cells play a crucial role in bacterial clearance by expressing the enzyme heme oxygenase 1 (Hmox1), which catalyzes the degradation of heme groups into Fe^2+^, biliverdin, and carbon monoxide (CO). The role of Hmox1 activity during *S.* Typhimurium infection is not clear and previous studies have shown contradictory results. We evaluated the effect of pharmacologic modulation of Hmox1 in a mouse model of acute and persistent *S.* Typhimurium infection by administering the Hmox1 activity inductor cobalt protoporphyrin-IX (CoPP) or inhibitor tin protoporphyrin-IX (SnPP) before infection. To evaluate the molecular mechanism involved, we measured the colocalization of *S.* Typhimurium and autophagosome and lysosomal markers in macrophages. Administering CoPP reduced the bacterial burden in organs of mice 5 days post-infection, while SnPP-treated mice showed bacterial loads similar to vehicle-treated mice. Furthermore, CoPP reduced bacterial loads when administered after infection in macrophages in vitro and in a persistent infection model of *S.* Typhimurium in vivo, while tin protoporphyrin-IX (SnPP) treatment resulted in a bacterial burden similar to vehicle-treated controls. However, we did not observe significant differences in co-localization of green fluorescent protein (GFP)-labeled *S.* Typhimurium with the autophagic vesicles marker microtubule-associated protein 1A/1B-light chain 3 (LC3) and the lysosomal marker lysosomal-associated membrane protein 1 (LAMP-1) in macrophages treated with CoPP. Our results suggest that CoPP can enhance antimicrobial activity in response to *Salmonella* infection, reducing bacterial dissemination and persistence in mice, in a CO and autophagy- independent manner.

## 1. Introduction

*S.* Typhimurium is a Gram-negative facultative anaerobic bacterium that causes foodborne illnesses in humans, with more than 1.2 million cases only in the United States [1]. The natural host for these bacteria can be poultry, swine, horses, cattle, wild rodents, and humans, in whom this pathogen causes gastroenteritis, fever, septicemia, and systemic disease [2]. *S.* Typhimurium is a major world health problem due to the increasing emergence of multidrug resistance cases over the last 30 years [3].

The ability of *Salmonella* to cause systemic diseases is due to its capacity to survive and replicate within phagocytic cells, evading the host immune response directed to clearing bacterial infection [4]. Once *Salmonella* is ingested and accesses the intestinal lumen, it invades epithelial cells and is then engulfed by macrophages and dendritic cells (DCs). Inside these cells, the bacteria can travel and spread to deeper organs, such as the spleen, liver, and lymph nodes [5,6]. Some studies have provided evidence of persistent *Salmonella* infections in mice various days post-infection and after antibiotic treatment [3]. This is an important feature of the *Salmonella*-caused disease, as it has been reported that persistent bacterial infection can lead to severe consequences, such as pancreatitis, among other chronic inflammatory diseases [7]. Our previous data showed that, after treatment with the antibiotic enrofloxacin, resident bacteria could be found in the spleen and liver 42 days post-infection in mice that did not produce the anti-inflammatory cytokine interleukin 10 (IL-10^−/−^ mice), a mouse model of Inflammatory Bowel Disease (IBD). We described that IL-10^−/−^ mice were less susceptible to the acute disease caused by *Salmonella* than WT IL-10-producing mice [6]. However, IL-10^−/−^ mice were more susceptible to developing chronic intestinal inflammation than uninfected control mice [8]. Importantly, in both WT and IL-10^−/−^ mice, persistent infections with *Salmonella* were observed after antibiotic treatment. This observation might have important clinical implications for disease progression, considering that antibiotics are used as part of the treatment in most *Salmonella* infected and IBD patients [8]. Moreover, another study described the persistence of *S.* Typhimurium in the cecum lymph node of infected mice, even after 10 days of ciprofloxacin treatment, which is a widely used antibiotic to treat salmonellosis in humans [9]. Furthermore, the presence of *S.* Typhimurium has been observed after nearly 3 months of infection in Gr1^+^ cells of the mesenteric lymph nodes of infected mice [10]. A study tracked the presence of *S.* Typhimurium by using fluorescent bacteria in mice, concluding that this bacterium would reside inside inducible nitric oxide synthase (iNOS)-producing macrophages, associated with a granuloma in the spleen, keeping bacteria away from T cells [11], which are in turn known to help resolving infection [6].

Considering that persistent infections with intracellular bacterial pathogens that cannot be cleared with many classes of antibiotics could lead to inflammatory disorders, such as chronic inflammation and/or future reactivation, it is relevant to identify natural bactericidal mechanisms of the host that could be boosted to eliminate persistent intracellular bacteria. Along these lines, it might be possible to achieve sterilization of the affected cells and tissues through the defense mechanisms of the host.

Among the molecules that are involved in the clearance of intracellular pathogens is heme oxygenase-1 (Hmox1). Hmox1 is an enzyme that catalyzes the first step of the oxidative degradation of the heme group, which is the rate-limiting reaction that releases carbon monoxide (CO), free iron (Fe^2+^), and biliverdin, which is rapidly reduced to bilirubin [12]. Hmox1 is composed of 288 residues and its active site is located between the first two alpha-helices of the protein [13]. Hmox1 is expressed in all mammalian tissues at basal undetectable levels, but in tissues where red blood cells or hemoglobin are degraded, such as the spleen, liver, bone marrow, and kidney, Hmox1 can be induced either by its substrate (heme), or drugs, such as cobalt protoporphyrin-IX (CoPP), and other physical and chemical stimuli [14,15,16,17].

Protoporphyrin-IX is an organic compound present in all cells, consisting of four pyrrole rings that frequently chelate metals to form metalloporphyrins, and it is a precursor of heme when chelates with iron by the mitochondrial enzyme ferrochelatase [18]. Metalloporphyrins are widely used as modulators of heme oxygenase (HO) activity in different models, for example, it has been described that cobalt protoporphyrin (CoPP) is an inducer of the expression of the gene that codes for the enzyme Hmox1 [19,20]. On the other hand, certain protoporphyrins such as tin protoporphyrin-IX (SnPP), and zinc protoporphyrin-IX (ZnPP) are used in various models to inhibit HO activity [21,22,23,24,25]. Although these protoporphyrins only differ by the cation in their structure, the opposite effect on HO activity resides in the ability of the enzyme to oxidize them. Maines and Kappas described in 1977 that CoPP not only binds to the active site of HO but is also oxidized, as is heme, resulting in an increased enzymatic activity. On the other hand, SnPP and ZnPP can bind to HO acting as competitive inhibitors and reducing the enzymatic activity [26,27,28].

Although little is known about the exact effect of Hmox1 on general bacterial infections, it has been shown that Hmox1 plays a role in the clearance of *Salmonella* by the immune system and that this effect is exerted by CO [29]. For instance, in an acute inflammation model, where C57BL/6 mice were treated with streptomycin before *S*. Typhimurium infection, CoPP treatment reduced the presence of *Salmonella* DNA in mesenteric lymph nodes, lamina propria, liver, and spleen three days post-infection [30]. This effect was explained by the release of CO as a result of Hmox1 activity, which may regulate the macrophage response to *S.* Typhimurium. Thus, in vitro depletion of Hmox1 in murine macrophages reduced their bactericidal capacity against *S.* Typhimurium, suggesting that the effect observed for CO was due to the capacity of this gas to promote bacterial clearance in phagocytic cells associated with the intestine [30]. An in vitro study of the bacterial clearance capacity of murine and human macrophages supports these findings. Specifically, it was observed that macrophages from Hmox1-deficient mice have bacterial killing defects that were restored by administering CO. In addition, CO treatment results in an enhanced bacterial clearance capacity in mouse and human macrophages. In contrast, CO does not affect the bactericidal activity of macrophages isolated from NALP3-deficient and caspase 3-deficient mice. Consequently, the bacteria-killing capacity of macrophages depends on CO-mediated inflammasome activation [31]. These results suggest that CO produced by Hmox1 activity has an antibacterial effect on macrophages. However, contradictory results were observed in murine macrophages transfected with a *Hmox1* shRNA. In these macrophages, intracellular *S.* Typhimurium survival was reduced upon *Hmox1* knockdown, an effect that was attributed to decreased iron availability inside the cell [32]. Therefore, reduced levels of *Hmox1* expression can also lead to increased clearance of intracellular *S.* Typhimurium.

Based on these reports that link *Hmox1* expression with intracellular *S.* Typhimurium clearance, we explored the role of Hmox1 activation in vivo during acute and persistent infections with this bacterium. We further evaluated whether pharmacological modulation of Hmox-1 can facilitate persistent bacterial clearance by the host. CoPP treatment resulted in reduced bacterial load in acute *S*. Typhimurium infection and no bacterial load in the persistent infection model. A similar reduction in *S.* Typhimurium survival in a macrophage cell line was observed in vitro when infected cells were treated with CoPP. As we approached elucidating the molecular mechanism involved, we found that the observed phenotype is independent of CO since treatment with a CO-releasing molecule (CORM3) did not result in a difference in bacterial survival within macrophages. Consequently, we evaluated if the autophagy pathway is involved. Autophagy is an intracellular strategy of self-digestion in which the cytoplasmic contents are enveloped in a double membrane vesicle, the autophagosome, which is delivered to the lysosome (in animal cells) to form the autolysosome, where its content will be subsequently degraded and recycled [33,34]. Cells can also use autophagy for the elimination of intracellular pathogens [35]; however, we did not see significant differences in co-localization of *S.* Typhimurium and themarkers of autophagic vesicles LC3 and LAMP-1, for autophagosomes and autolysosomes, respectively, between CoPP and vehicle-treated macrophages. Further work is needed to evaluate the exact molecular mechanism behind this phenomenon, however, our in vivo results strongly suggest the potential of the modulation of endogenous targets in the treatment of infectious diseases, such as *Salmonella* Typhimurium.

## 2. Materials and Methods

### 2.1. Mice

C57BL/6J female mice (6 to 8 weeks of age) were originally obtained from Jackson Laboratories and maintained at the specific pathogen-free central facility of the Pontificia Universidad Católica de Chile in ventilated racks. All animal work was reviewed and approved by the Scientific Ethical Committee for Animal and Environment Care of the Pontificia Universidad Católica de Chile and the Scientific Committee for Research Biosafety (Protocol number 170721004). Experiments were performed and conducted in agreement with institutional and international Guidelines for Animal Care. The number of animals per group was calculated using G*Power 3.1.9.2 software. Using previous data on bacterial loads in mice from our laboratory, the software calculated the minimum number of mice needed to observe significant differences.

### 2.2. In Vivo Infection and Monitoring

Mice were anesthetized by inhalation of isoflurane at 2% (1 L/min) and inoculated with 1 × 10^6^ colony forming units (CFU) of wild-type *S.* Typhimurium by intragastric gavage, using an intravenous catheter (20 GA × 1.88″). In the case of acute infections, mice were monitored to measure weight loss and clinical scores daily for 5 days until euthanasia. For persistent infection, mice were monitored to measure weight loss and clinical score daily for 6 days, and then every other day until day 42 post-infection, when mice were euthanized (Table A1).

### 2.3. Bacterial Strain and Infective Dose Preparation

Wild-type *S.* Typhimurium 14028 was obtained from the American Type Culture Collection (ATCC) and kindly provided by Dr. Carlos Santiviago (Universidad de Chile, Santiago, Chile). *S.* Typhimurium pkk233.2 GFP::Amp (*S*. Typhimurium-GFP) was provided by Dr. Alexis Kalergis, Faculty of Biological Sciences, Pontificia Universidad Católica de Chile Stocks of frozen bacteria were stored at −80 °C in glycerol 20% in the Cryobank system. For each infection experiment (in vitro and in vivo), one bead of the tube with *S.* Typhimurium stock was grown in LB broth or LB medium with ampicillin (100 µg/mL) in agitation at 37 °C overnight (ON). Then a subculture was done in LB broth 1:1000 until OD_600_ 0.6. Depending on the exact OD that was reached, the volume with the CFU needed for infection of mice or cells was calculated. This volume was centrifuged at 6200× *g* for 10 min at 4 °C and resuspended in sterile phosphate buffered saline (PBS). To verify the dose of infection, the inoculum was seeded in LB Agar or LB Ampicillin Agar plates in serial dilutions.

### 2.4. CoPP and SnPP Preparation

Cobalt protoporphyrin-IX and tin protoporphyrin-IX (Sigma-Aldrich, St. Louis, MO, USA) were diluted in NaOH 1 N at 10 mM. This stock was stored at 4 °C in amber tubes. The volume needed for each animal was calculated based on the weight of the mouse (5 mg/kg for both drugs) and diluted in 150 µL of sterile PBS per dose. The control vehicle was prepared by replacing the volume of the drug for NaOH 1 N diluted in 150 µL of sterile PBS per dose. The administration of the drugs was performed by intraperitoneal (ip) injection using an insulin syringe (BD Ultra Fine, Franklin Lakes, NJ, USA).

### 2.5. Enrofloxacin Administration

Enrofloxacin 10% (Enromic^®^ 10%, Centrovet, Santiago, Chile) was administered to mice through drinking water at 2 mg/mL, from day 3 post-infection for 28 days. The bottle of water with Enrofloxacin was replaced every other day during the time of the treatment.

### 2.6. Bacterial Loads in Organs and Tissues

Organs and tissues were weighed once extracted from the mouse and placed in sterile PBS. Then, the organs were disrupted with a 70 µm cell strainer. In the case of feces samples, these were disaggregated in 1.5 mL tubes with autoclaved plastic pistils. The samples were diluted serially six times in sterile PBS and one drop of 10 µL of each dilution was seeded in triplicates in LB Agar (Difco, BD, Franklin Lakes, NJ, USA) for the liver and spleen, and one drop of 5 µL of each dilution was seeded in triplicates in MacConkey Agar (Difco, BD, Franklin Lakes, NJ, USA) for mesenteric lymph nodes and blood, or *Salmonella*-*Shigella* Agar (Difco, BD) for feces. Agar plates were incubated at 37° for approximately 16 h.

### 2.7. RNA Extraction

RNA extraction from tissues was performed using Trizol Reagent (Invitrogen, Waltham, MA, USA) following the protocol recommended by the manufacturer. Organs were homogenized in 1 to 3 mL depending on the size of the organ, 200 µL of chloroform (Merck, Darmstadt, Germany) were added, and then the sample was mixed by agitation for 15 s and incubated at room temperature (RT) for 2–3 min. Next, the samples were centrifuged at 13,000× *g* for 15 min at 4 °C. The aqueous phase was transferred to a new tube and 500 µL of Isopropanol was added and the samples were incubated for 30 min at −20 °C. Then, the tubes were centrifuged at 12,000× *g* for 10 min at 4 °C and the supernatant was eliminated. A measure of 1 mL of 75% Ethanol was added to the pellet and the tubes were centrifuged at 21,000× *g* for 5 min at 4 °C. The supernatant was eliminated again, and the pellet was air-dried for 5 min. The pellet was then diluted in nuclease-free (NF) water, and the samples were measured in Nanodrop to obtain RNA concentration and purity.

### 2.8. Quantitative Real-Time PCR

Quantitative real-time PCR (qPCR) was performed to detect transcription of genes encoding *Hmox1*, *IL-10*, *IFN-γ*, and *IL-1β*, using Taqman RNA-to-Ct 1-Step kit (Applied Biosystems, Waltham, MA, USA), following the manufacturer’s instructions for a 10 μL reaction. Hmox1 and cytokines transcriptions were normalized with β-2-microglobulin (B2M) transcription. Thermo Fisher ID for probes and primers used: Hmox1 Mm00516005_m1; IL-10 Mm00439614_m1; IFN-γ Mm01168134_m1; IL-1β Mm00434228_m1; and B2M Mm00437762_m1.

### 2.9. Western Blot

Western blot analyses were performed to assess the expression of HMOX-1. Protein preparations from the liver and spleen were extracted using a RIPA (radioimmunoprecipitation assay) protein extraction buffer after being frozen in liquid nitrogen immediately after extraction from mice. Proteins in the soluble fraction were then quantified using the Pierce BCA Protein Assay Kit (Thermo Fisher Scientific, Waltham, MA, USA). A measure of 50 µg of protein were loaded onto sodium dodecyl sulphate-polyacrilamide gel electrophoresis (SDS-PAGE) polyacrylamide 15% gels (Miniprotean II, BIO-RAD Laboratories, Hercules, CA, USA) and transferred onto 0.45 µm nitrocellulose membranes (BIO-RAD, Hercules, CA, USA). After transfer, membranes were blocked with milk 5% in tris buffered saline (TBS)-Tween and incubated, either with Hmox1 monoclonal antibody (HO-1-1, Invitrogen, MA1-112) at a dilution of 1:1000 at 4 ºC ON or an anti-β-actin antibody (Biolegend, clone 2F1-1) at a dilution of 1:1000 at 4 ºC ON in 5% bovine serum albumin (BSA) TBS-Tween. After incubation, the membranes were washed three times with TBS-Tween 0.01% (ChemCruz, Dallas, TX, USA) and incubated with Goat Anti-Mouse IgG (H + L)-HRP Conjugate, (BIO-RAD, Hercules, CA, USA, Cat 1706516) for 45 min at room temperature at a dilution of 1:5,000. After incubation with the secondary antibody, membranes were washed three times with TBS-Tween 0.01% and incubated with luminol:coumaric acid solution to detect membrane-bounded antibodies. Quimioluminiscence derived from this reaction was visualized using a ChemiDoc Imaging System (BIO-RAD, Hercules, CA, USA).

### 2.10. Ferritin ELISA

Ferritin was quantified from mouse serum using the Ferritin Mouse ELISA kit (Abcam) as per the manufacturer’s specifications. Serum samples were diluted at 1:40 and 1:200 and tested in duplicate. Ferritin concentration in each sample was calculated based on the standard curve determined for each plate.

### 2.11. Cell Culture, Treatment and Infection

A vial of RAW264.7 cells, provided by Dr. Leandro Carreño of the Instituto de Ciencias Biomédicas, Facultad de Medicina, Universidad de Chile, was used. The vial was expanded using Dulbecco’s Modified Eagle Medium (DMEM) medium (Gibco, Waltham, MA, USA), pH 7.2 and supplemented with 10% (*v*/*v*) fetal bovine serum (FBS). Cells were left in 75 cm^2^ culture bottles and allowed to differentiate at 37 °C with 5% CO_2_. Every two days the DMEM FBS 10% medium was replaced with a fresh medium. After obtaining the full confluence of the culture bottle, 4 × 10^5^ cells per well were distributed in a 24-well ELISA plate. Cells were infected with *S.* Typhimurium-GFP at a multiplicity of infection (MOI) equal to 25, centrifuged at 2000 rpm for 3 min and incubated for 1 h at 37 °C with 5% CO_2_. Then, the cells were treated with 100 µg/mL gentamicin for 1 h to kill extracellular bacteria and the different treatments were applied to each well: CoPP (50 μM, Sigma-Aldrich, St. Louis, MO, USA), SnPP (50 μM, Sigma-Aldrich, St. Louis, MO, USA), CORM3 (100–200 μM, Sigma-Aldrich, St. Louis, MO, USA), inactivated CO-releasing molecule (iCORM3) (37 °C with 5% CO_2_ ON before to the experiment), NaOH (0.75 mM, CoPP vehicle), and dH_2_O (CORM3 vehicle). Cells were then incubated for 4 h at 37 °C and prepared for flow cytometry analysis.

### 2.12. Flow Cytometry

Following treatment of RAW264.7 cells for 4 h with the respective drugs, the medium was discarded and 200 µL of PBS was added. The bottom of the well was scraped with a pipette tip and the contents were transferred to a 96-well plate. The plate was centrifuged at 2000 rpm for 5 min and the cells were stained with 50 µL of the viability probe “BD horizon fixable viability stain 510” (dilution 1:1000) for 15 min at 4 °C, cells positive for this marker are defined as dead, then washed with 50 µL PBS and centrifuged again at 2000 rpm for 5 min. Samples were fixed with 200 µL paraformaldehyde (PFA) for 10 min, centrifuged again at 2000 rpm for 5 min and 150 µL PBS was added to resuspend the cells. Data were acquired on a Fortessa X-20 flow cytometer (BD Bioscience, Oxford, UK) and analyzed using FlowJo software v10.8.1 (Tree Star, Ashland, OR, USA).

### 2.13. Electron Microscopy

RAW264.7 cells were infected with *Salmonella* ser. Typhimurium-GFP at a multiplicity of infection (MOI) equal to 25 for 4 h. After infection, the bottom of the well was carefully scraped with a cell scraper and the contents were transferred to a 1.5 mL eppendorf tube. The samples were centrifuged at 1500 rpm for 10 min and the supernatant was then carefully discarded so as not to move the cell pellet. Subsequently, cell pellets were fixed in 2.5% glutaraldehyde in 0.1 M sodium cacodylate buffer pH7.0 for 6 h at room temperature and washed in cacodylate buffer ON at 4 °C. Cells were then fixed in 1% aqueous osmium tetroxide for 90 min and washed with double distilled water 3 times for 15 min each. Then, *en bloc* staining was performed with 2% aqueous uranyl acetate for 60 min and then dehydrated with 50, 70, 95, 95, 100 and 100% acetone battery for 15 min each. Samples were left ON in resin:acetone 1:1 and then in pure resin for 4 h. The pure resin was changed and polymerized in the incubator at 60 °C for 48 h. Thin sections were obtained in Leica Ultracut R ultramicrotome and stained with 4% uranyl acetate in methanol for 2 min and with lead citrate for 5 min. Samples were observed in a Philips Tecnai 12 transmission microscope at 80 kV (Advanced Microscopy Unit, Facultad de Ciencias Biológicas, Pontificia Universidad Católica de Chile).

### 2.14. Immunofluorescence and Fluorescence Microscopy

RAW264.7 cells were infected, and the different treatments were applied to each well: CoPP (50 μM), SnPP (50 μM) and NaOH (0.75 mM). Cells were treated with Bafilomycin (100 nM, autophagic flux inhibitor) and Rapamycin (1 µM, autophagy inducer) as controls [36]. Following treatments, cells were fixed with 4% (*w*:*v*) PFA for 20 min at room temperature. Cells were then permeabilized with PBS-triton 0.1% for 10 min at room temperature, blocked with PBS-BSA 3% at room temperature for 1 h and incubated ON with the following primary antibodies at 4 °C: LC3 (1:250; PM036, MBL), LAMP1 (1:500; 553792, BD Pharmingen, USA). Staining of the primary antibodies was followed by conjugation with their respective secondary antibody (1:300; Alexa Fluor^®^, Life Technologies, Carlsbad, CA, USA) for 1 h at room temperature. Nuclei were counterstained with DAPI for 10 min at dark and room temperature. Images were taken on an inverted fluorescence microscope (LSM 880 ZEISS with Airyscan detection, Advanced Microscopy Unit, Facultad de Ciencias Biológicas, Pontificia Universidad Católica de Chile).

### 2.15. Statistical Analysis

All statistical analysis, including one-way ANOVA, 2-way ANOVA, Student’s *t*-test and chi-square were performed using GraphPad Prism 9 version 9.0.1. The test used and the value of *p* or chi-square for each experiment are detailed in the caption of the figures.

## 3. Results

### 3.1. CoPP-Mediated Hmox1 Induction Does Not Influence an Acute S. Typhimurium Infection

Previous studies have shown that Hmox1 has a role in *S.* Typhimurium clearance after 72 h of infection [30]. To evaluate whether this phenomenon is also observed during an acute systemic model of infection, we treated C57BL/6J female mice with 5 mg/kg CoPP and the Hmox1 competitive inhibitor tin SnPP, and 24 h later, mice were infected with 1 × 10^6^ CFU of *S.* Typhimurium. Clinical scores and survival rates of animals were monitored for fourteen days post-infection. Briefly, mice clinical scores include weight, posture, and response to stimuli. These parameters were measured daily until the end of the experiments. As described in Table A1, the sum of the score is registered and euthanasia is considered in cases of scores equal to or higher than seven.

**Figure 1:**
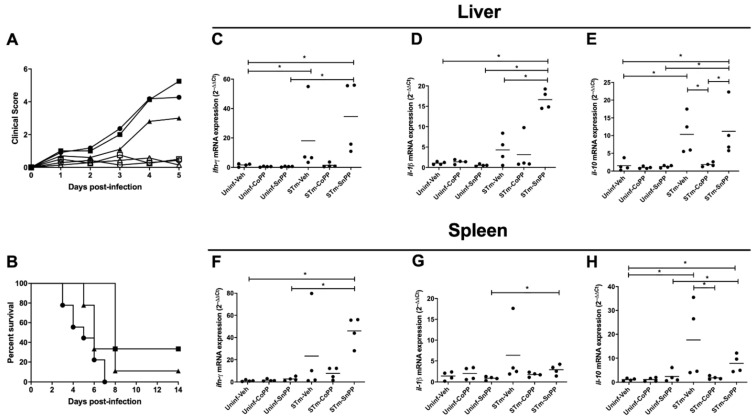
Hmox1 induction has no major effect on the clinical score and survival rates and has a mild anti-inflammatory effect in the acute model of *S*. Typhimurium infection in mice. Female C57BL/6 mice were treated with CoPP, SnPP, or vehicle (Veh) 24 h before intragastric infection with 1 × 10^6^ CFU of *S.* Typhimurium (STm) or PBS as negative control (Uninf). (**A**) Clinical score was evaluated in infected and uninfected mice with all the treatments until day 5 post-infection or PBS administration (Uninf-Veh: white circles; Uninf-CoPP: white squares; Uninf-SnPP: white triangles; STm-Veh: black circles; STm-CoPP: black squares; STm-SnPP: black triangles (**B**) survival rates were evaluated daily until day 14 post-infection (STm-Veh n = 11; STm-CoPP n = 10; STm-SnPP n = 8; 3 independent experiments). No significant differences were observed between treatments. A 2-way ANOVA was used to compare differences between days in the same treatment. Mice were euthanized on day 5 post-infection and quantitative real-time PCR (qPCR) was performed to detect transcription of genes encoding IFN-γ, IL-1β and IL-10 in the liver and spleen (**C**–**H**) (n = 4, 1 independent experiment). The Student’s *t*-test was used to evaluate differences, and results with statistically significant differences are shown (*: *p* < 0.05).

As shown in Figure 1, we observed that CoPP treatment tends to reduce clinical score when compared with SnPP-treated and vehicle-treated mice, although no significant differences were found between treatments (Figure 1A). Survival assessment also showed that both CoPP and SnPP treatments slightly increased the survival of the infected mice, although no statistically significant differences were observed (Figure 1B). These results suggest that modulation of Hmox1 through a single pharmacological induction during an acute *S*. Typhimurium infection has no significant effect on the disease caused by this bacterium in mice.

Because pro-inflammatory cytokine production is a parameter used to measure the inflammation caused by infections in tissues, we measured transcription of the pro-inflammatory cytokines *ifn-γ* and *il-1β* mRNA by qRT-PCR in the liver and spleen of infected mice that were treated either with CoPP, SnPP or vehicle. Due to the dispersion of the data obtained for each group, no significant differences were found. Nevertheless, we observed a tendency in CoPP-treated mice to reduce pro-inflammatory cytokines in the liver (Figure 1C,D) and spleen (Figure 1F,G), when compared with vehicle or SnPP-treated mice. These results may indicate that, in this model, CoPP could have either a mild anti-inflammatory effect or a preventive effect in *S*. Typhimurium dissemination.

Previous studies from our laboratory have shown that acute *S.* Typhimurium infection induces the production of high levels of IL-10 5 days post-infection in the liver and spleen, which correlates with infection severity [6]. A similar result was observed in vehicle-treated and SnPP-treated mice, but less transcription of IL-10 was observed in the liver (Figure 1E) and spleen (Figure 1H) of CoPP-treated mice. This result suggests that CoPP treatment may limit the systemic spread of *S*. Typhimurium, which results in reduced IL-10 production.

### 3.2. Hmox1 Induction by CoPP Treatment Reduces Bacteria Load in Tissues but Not in the Blood of S. Typhimurium-Infected Mice

**Figure 2:**
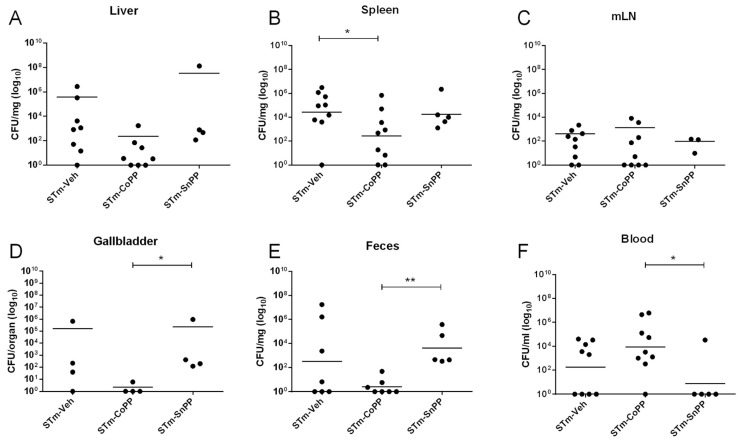
Hmox1 induction with CoPP treatment reduces bacterial loads in an acute *S.* Typhimurium infection model in mice. Female C57BL/6 mice were treated with CoPP, SnPP, or vehicle 24 h before intragastric infection with 1 × 10^6^ CFU of *S.* Typhimurium. Mice were euthanized on day 5 post-infection and bacterial loads in (**A**) liver, (**B**) spleen, (**C**) mesenteric lymph nodes, (**D**) gallbladder, (**E**) feces and (**F**) blood were measured. Geometric means are shown (STm-Veh n = 11; STm-CoPP n = 10; STm-SnPP n = 8; 3 independent experiments). The Student’s *t*-test was used to evaluate differences, and results with statistically significant differences are shown (*: *p* < 0.05; **: *p* = 0.005).

**Figure A1:**
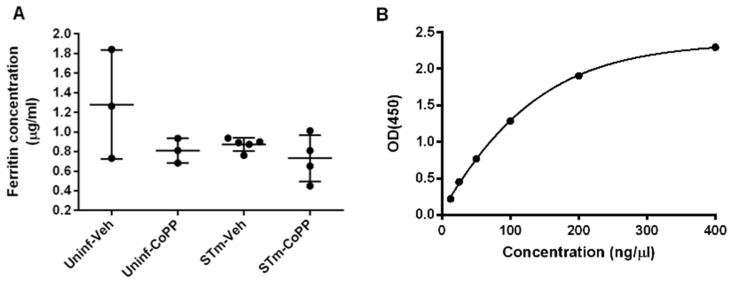
Ferritin levels evaluated by ELISA in sera of *S.* Typhimurium-infected mice untreated or CoPP-treated. (**A**). Female C57BL/6 mice were treated with CoPP or vehicle as uninfected controls. Twenty-four h later, mice were intragastrically infected with 1 × 10^6^ CFU of *S*. Typhimurium 14028 and on day 6 post-treatment (day 5 post-infection of infected groups) serum was obtained to measure Ferritin using the Ferritin Mouse ELISA kit (Abcam). Serum samples were diluted at 1:40 and 1:200 and tested in duplicate. (**B**). Ferritin concentration in each sample was calculated based on the standard curve determined for each plate. No significant differences were observed.

C57BL/6J female mice were treated as described above with 5 mg/kg CoPP and 5 mg/kg SnPP. Twenty-four hours later, mice were infected with 1 × 10^6^ CFU of *S.* Typhimurium, and after 5 days of infection, mice were euthanized to evaluate bacterial burden in organs. As shown in Figure 2, reduced bacterial loads were observed in spleen, gallbladder, and feces derived from CoPP-treated mice, when compared with vehicle- or SnPP-treated mice, suggesting a role of Hmox1 in bacterial clearance. However, the opposite result was observed in blood, where CoPP-treated mice displayed higher bacterial loads when compared with the other experimental groups. To evaluate possible explanations for this result, we measured ferritin by ELISA in serum from mice, as an indirect indicator of the amount of iron. However, we did not observe significant differences between any of the groups (Figure A1).

### 3.3. Prophylactic Hmox1 Induction by CoPP Reduces the Persistence of S. Typhimurium in Mice

**Figure 3:**
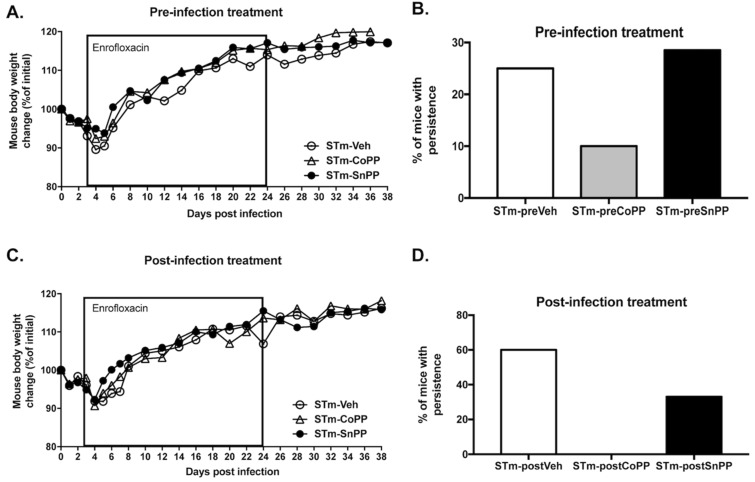
Hmox1 induction by CoPP treatment reduces persistent *S.* Typhimurium infection model in mice. Female C57BL/6 mice were treated with CoPP, SnPP, or vehicle 24 h before (**A**,**C**) or 72 h after (**B**,**D**) intragastric infection with 1 × 10^6^ CFU of *S.* Typhimurium. In both cases, Enrofloxacin was administered in the drinking water from day 3 to day 24, to allow mice to survive to infection and promote bacterial persistence until the end of the experiment. On day 38 post-infection, mice were euthanized and bacterial loads were measured in organs and blood. (**A**,**C**) Weight change is represented as a percentage of initial weight. (**B**,**D**) Percentage of mice with persistent infection at 38 days post-infection in at least one organ/blood (STm-preVeh n = 8; STm-preCoPP n = 10; STm-preSnPP n = 14; STm-postVeh = 5; STm-postCoPP = 6; STm-postSnPP n = 12; 3 independent experiments). No significant differences were observed in the weight change of mice. Prospective analysis was performed: for B: chi-square = 10.30 and *p* = 0.0055; for D: chi-square = 84.34 and *p* < 0.0001.

**Figure A2:**
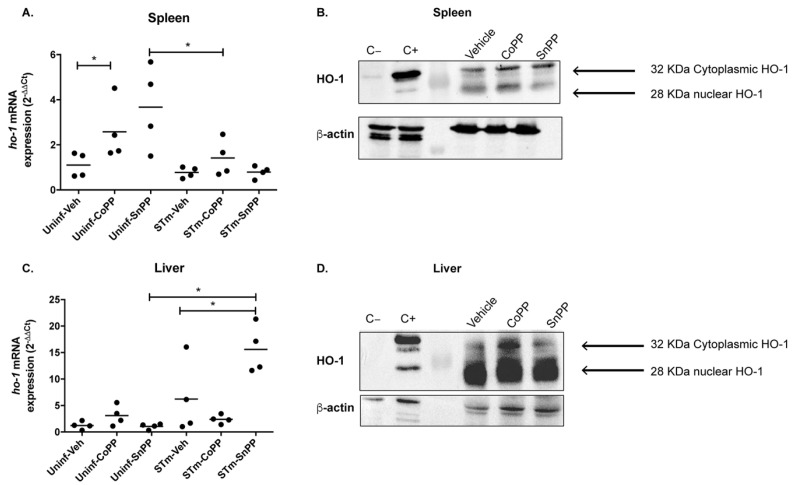
Evaluation of *Hmox-1* transcription in tissues of *S.* Typhimurium-infected mice. Female C57BL/6 mice were treated with CoPP, SnPP or vehicle as uninfected controls. Twenty-four hours later, mice were intragastrically infected with 1 × 10^6^ CFUs of *S.* Typhimurium and on day 6 post-treatment (day 5 post-infection of infected groups), the liver and spleen were recovered to purify RNA or proteins. (**A**,**C**) Quantitative real time PCR (qPCR) was performed to detect transcription of genes coding for Hmox-1, using the indicated probes in Materials and Methods. As a housekeeping gene, quantification of beta-2-microglobulin mRNA was performed. Data are expressed as fold-increase in DDCt. Student’s *t*-test was used to evaluate differences. Results with statistically significant differences are shown (*: *p* < 0.05). (**B**,**D**). Representative images of Western blot analyses performed to assess the production of Hmox-1. Protein preparations from the liver and spleen were performed using RIPA protein extraction buffer and a Western blot assay was performed using anti-Hmox1 and anti-β-actin antibodies. SH-SY5Y cells were treated for 14 h with 10 µM SnPP (negative control) or 10 µM CoPP (positive control).

To evaluate whether Hmox1 induction through CoPP treatment can modulate *S.* Typhimurium persistent infection, mice were treated with CoPP 5 mg/kg, SnPP 5 mg/kg or vehicle intraperitoneal (ip) 24 h before infection with 1 × 10^6^ CFU of *S.* Typhimurium. The treatment was then repeated once a week. Due to the severity of this infection, mice were treated after 3 days of infection with the antibiotic Enrofloxacin in the drinking water for 3 weeks, to allow mice to survive until the end of the experiment. Clinical parameters and survival proportions were measured until the end of the experiment on day 38 post-infection. Weight loss was not significantly different between each of the three groups of mice (Figure 3A). Bacterial loads from the spleen, liver, lymph nodes and blood were measured at day 38 post-infection by plating homogenized tissues in agar plates (Table 1). With this information, a prospective analysis was performed, revealing a significant difference (*p* = 0.0055) in persistence in at least one organ/blood between CoPP-treated mice and the other two groups (Figure 3B). These results indicate that a consistent state of high expression of Hmox1 modulates the persistent infection of *S.* Typhimurium in mice. It is important to mention that Hmox1 protein expression was corroborated by western blot in the spleen and liver, although the induction of the protein was only observed in the liver (Figure A2)**.**

### 3.4. Hmox1 Induction by CoPP Treatment Reduces the Persistence of S. Typhimurium When Administrated Post-Infection

To further evaluate the beneficial effect of Hmox1 in bacterial persistence, we treated mice after the beginning of the infection using the drug as a post-disease treatment. Then mice were infected with 1 × 10^6^ CFU of *S.* Typhimurium and 3 days later were injected ip with CoPP 5 mg/kg, SnPP 5 mg/kg, or vehicle. On the same day, enrofloxacin treatment began. Clinical parameters and survival proportions were measured until the end of the experiment on day 38 post-infection. As observed in the prophylactic treatment, weight loss was not statistically different (Figure 3C). Bacterial loads from the spleen, liver, lymph nodes, and blood were measured at day 38 post-infection by seeding homogenized tissues in agar plates (Table 2). A prospective analysis was used as described above, and significant differences (*p* < 0.0001) were observed in persistence between the groups (Figure 3D), indicating that CoPP can be effectively used as a treatment to reduce bacteria persistence when the treatment is started after the onset of the infection.

### 3.5. CoPP and SnPP Treatments Modulate S. Typhimurium Survival in RAW264.7 Cells in a CO-Independent Manner

**Figure 4:**
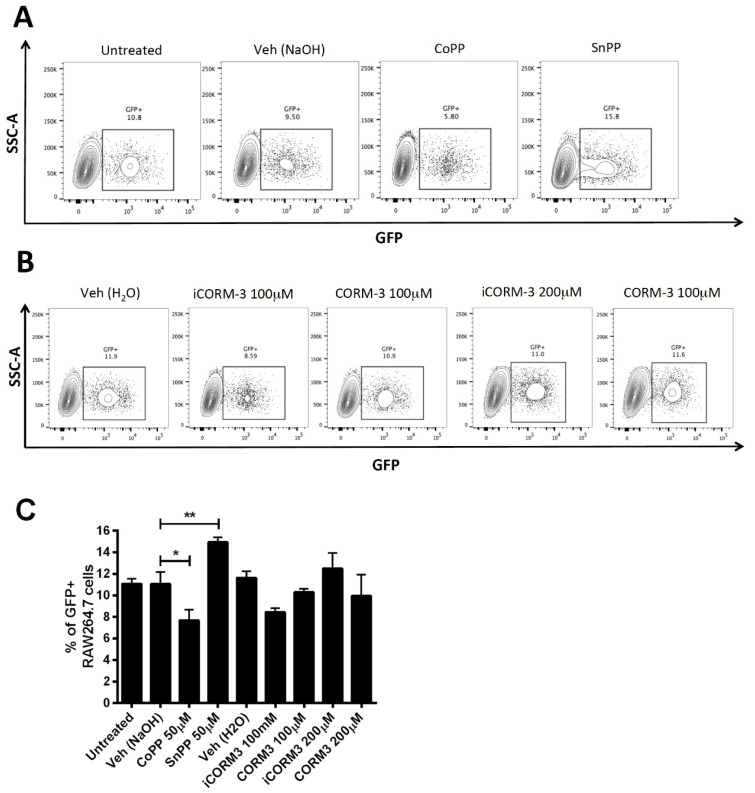
CoPP and SnPP treatment modulate *S.* Typhimurium survival in RAW264.7 cells in a CO-independent manner. RAW264.7 cells were infected with *S.* Typhimurium-GFP at MOI 25 for 1 h and treated with 100 µg/mL gentamicin for 1 h to kill extracellular bacteria. Cells were then treated with either vehicle (0.75 mM NaOH for CoPP and SnPP and dH_2_O for CORM3), CoPP 50 μM, SnPP 50 µM, CORM3 100 or 200 μM, or inactive CORM3 for 4 h. Cells were stained with a viability dye for flow cytometric analysis. (**A**,**B**). Representative dot plots are shown for each treatment. Results are presented as a percentage of live cells. (**C**). Mean of percentages for each treatment in two independent experiments. Differences were evaluated with one-way ANOVA, and significant ones are shown (*: *p* < 0.05; **: *p* < 0.005).

**Figure A3:**
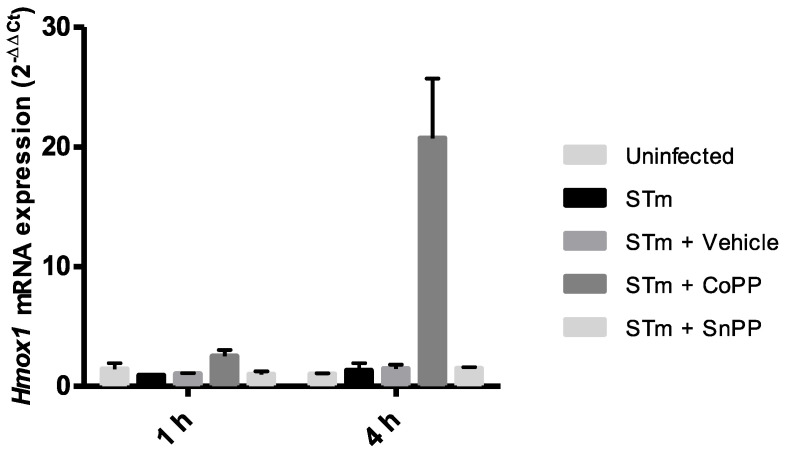
Relative expression of *hmox1* in RAW264.7-infected cells treated with Hmox1 modulating drugs. RAW264.7 cells were infected with *S.* Typhimurium MOI 25 for 1 h, treated with 100 µg/mL gentamicin for 1 h and then treated with either vehicle (0.75 mM NaOH), CoPP 50 μM, SnPP 50 μM for 1 and 4 h.

To replicate the phenotype observed in mice in an in vitro model, we evaluated the effect of CoPP and SnPP treatment on the survival of *S.* Typhimurium in a macrophage cell line. We infected RAW264.7 cells with a strain of *S.* Typhimurium that contains a plasmid coding for the green fluorescent protein (GFP) using a multiplicity of infection (MOI) of 25, for 1 h. Then, cells were treated with Gentamicin for 1 h to eliminate all extracellular bacteria and then treated with vehicle, CoPP 50 µM, or SnPP 50 µM for 4 h. This time was selected by measuring *hmox1* induction in the same conditions to corroborate that the treatment was effective (Figure A3). The survival of bacteria was evaluated by flow cytometry. As we expected, CoPP treatment significantly reduced bacterial survival inside cells compared with no treatment (Figure 4A,C). In contrast, SnPP treatment resulted in a significant increase in bacterial survival, as compared with vehicle-treated cells. Therefore, these results confirm that CoPP decreases *S.* Typhimurium survival in macrophages, whereas SnPP induces the opposite effect.

Carbon monoxide (CO) has been proposed as the responsible for the bactericidal effect of Hmox1 in several bacterial infection models [37,38,39] and is even considered a therapy [40]. To evaluate if the phenotype observed in our in vivo and in vitro results is due to the CO produced in the HO reaction, we treated RAW264.7 cells with CO-releasing molecule 3 (CORM3). Using the same experimental design described above, we treated cells with CORM3 100 µM, 200 µM or its inactivated form iCORM3. No significant differences were observed among the concentrations used (Figure 4B,C), suggesting that the effect of Hmox1 observed is CO-independent.

**Figure 5:**
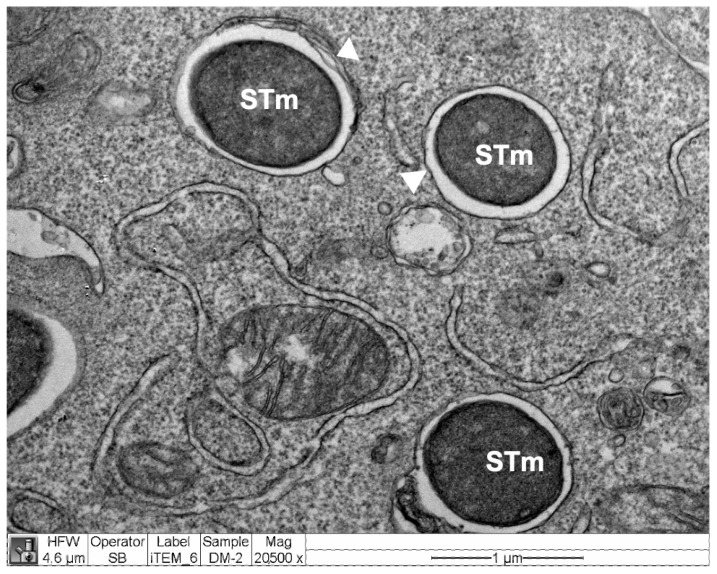
*S*. Typhimurium-GFP is found inside autophagosomes in RAW264.7 cells. RAW264.7 cells were infected with STm-GFP for 4 h. Arrowheads indicate a double-membrane belonging to the autophagosome surrounding bacteria (STm). Representative of one individual experiment.

To explore alternative mechanisms, and given the important role played by antibacterial autophagy in restricting intracellular bacterial replication [35], we sought to determine whether autophagy could be the mechanism involved in the elimination of *S.* Typhimurium by Hmox1 induction. Figure 5 shows the presence of *S.* Typhimurium inside double-membrane structures corresponding to autophagosomes, in RAW264.7 cells, suggesting that these cells use the autophagic pathway to restrict *S.* Typhimurium infection, as has been observed in other cellular models [41,42].

**Figure 6:**
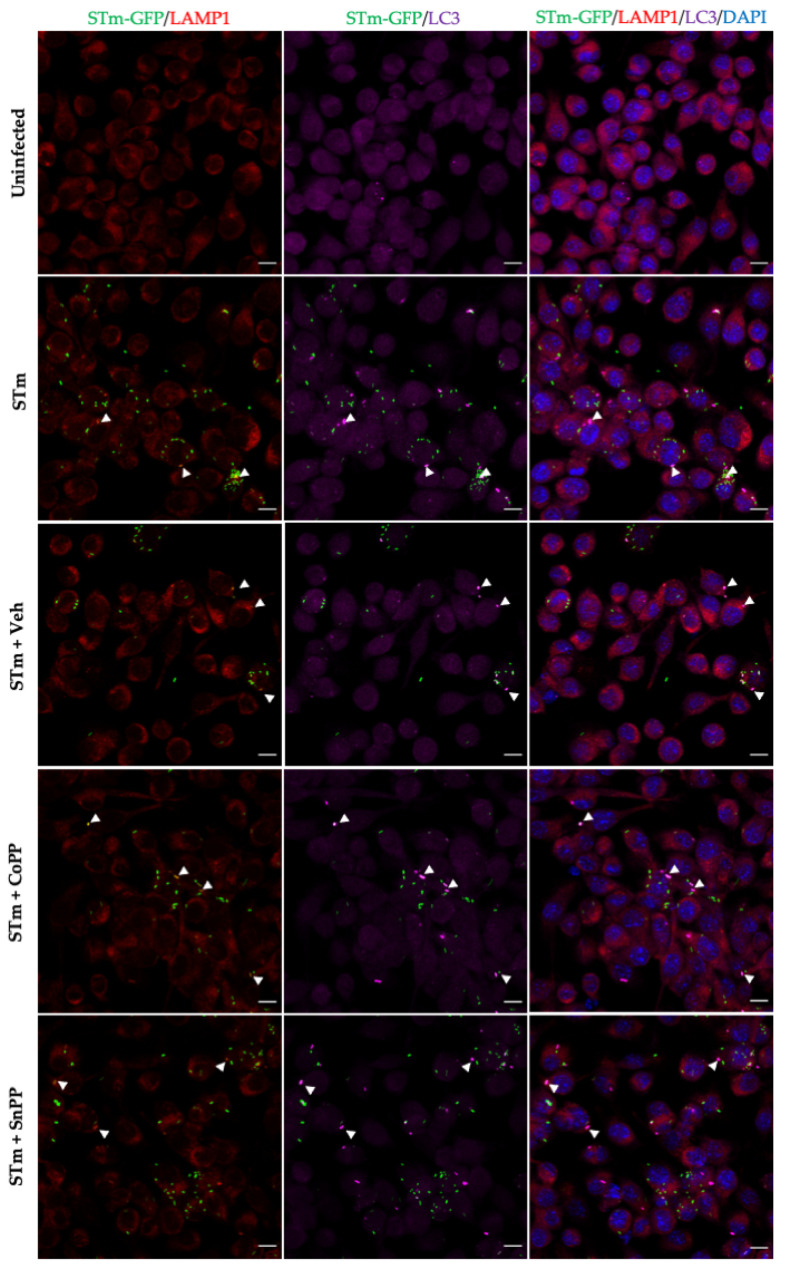
Co-localization of *S*. Typhimurium-GFP with autophagosome and lysosome markers in infected RAW264.7 cells. RAW264.7 cells were infected with STm-GFP for 1 h and subsequently treated for 4 h in the presence of vehicle (NaOH), CoPP, or SnPP. LC3: red; LAMP-1; magenta; DAPI: blue. STm-GFP colocalization with both markers is shown in white arrowheads. Scale Bar: 10µm. Images are representative of two independent experiments.

**Figure 7:**
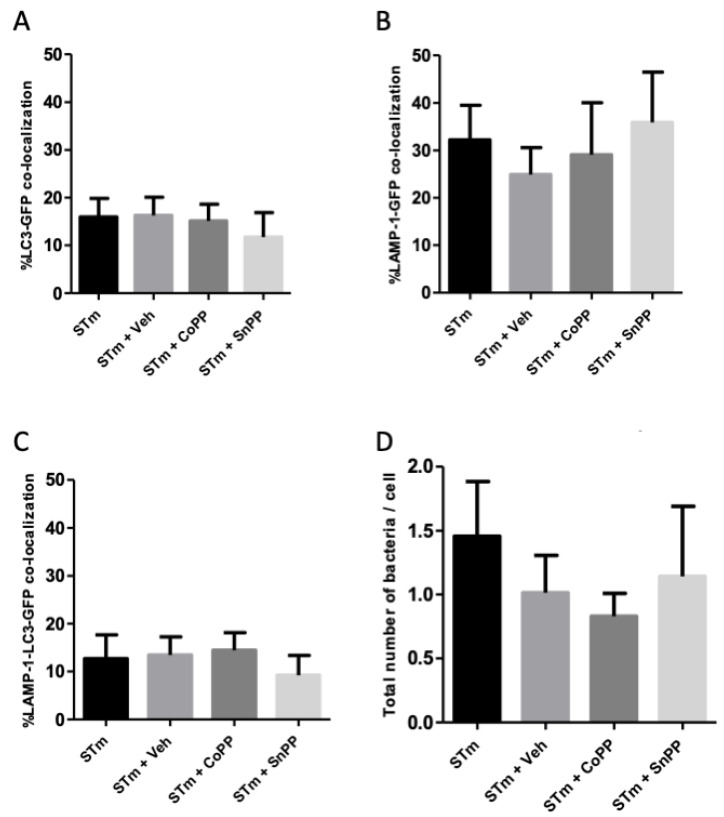
Quantification of colocalization of *S*. Typhimurium with autophagosome and lysosome markers in infected RAW264.7 cells. Microscopy images represented in Figure 6 were analyzed with ImageJ software. (**A**) Percentage of colocalization of *S*. Typhimurium-GFP with autophagosome (LC3). (**B**) Percentage of colocalization of *S*. Typhimurium-GFP with lysosomes (LAMP-1). (**C**) Percentage of colocalization of *S*. Typhimurium-GFP with autolysosomes (LC3 + LAMP-1). (**D**) Number of *S*. Typhimurium-GFP per cell. Representative of two independent experiments.

**Figure A4:**
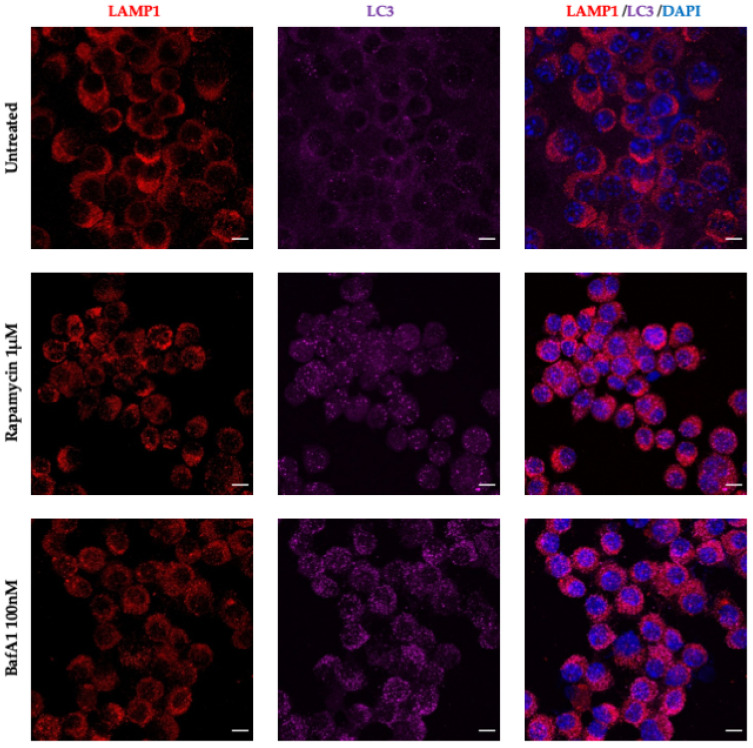
Positive and negative controls for autophagy. Cells were treated with the autophagy inductor Rapamycin 1 µM for 2 h, and with the autophagy inhibitor Bafilomycin A1 (BafA1) 100 nM for 4 h. Bars, 10 µm.

The next step in determining whether autophagy might be the mechanism involved was to perform immunofluorescence assays. A classical marker of autophagic structures is LC3 [36]. To corroborate what was observed in electron microscopy (Figure 5), we used LC3, as an autophagosome marker, and LAMP-1, as a lysosomal marker. RAW264.7 cells were infected with *S*. Typhimurium-GFP for 1 h and subsequently treated for 4 h in the presence of CoPP, SnPP, or vehicle (NaOH). The results obtained by fluorescence microscopy are shown in Figure 6. We observed that CoPP-treated RAW264.7 cells show no change in co-localization of *S*. Typhimurium-GFP together with LC3 and LAMP-1 (Figure 6 STm + CoPP, co-localization shown in white arrowheads) compared to infected vehicle-treated cells. Interestingly, it is possible to visualize that bacteria co-localized with these markers had lower GFP signal intensity or even no signal at all, suggesting that the bacteria are located within autolysosomes, which exhibit low pH that may affect bacterial fluorescence emission [36,43]. SnPP-treated cells also show some degree of co-localization, but it is only a proportion of the bacteria-GFP, and this could also be explained by increased bacterial survival, which may increase the possibility of autophagy (Figure 6 STm + SnPP, co-localization shown in white arrowheads). Of note, positive and negative controls, rapamycin and bafilomycin, respectively, were used (Figure A4). Multiple of these images obtained were analyzed. The percentage of *S.* Typhimurium-GFP co-localizing with LC3, LAMP-1, and both markers are presented in Figure 7. In addition, the number of bacteria per cell present in each of the treatments was quantified. The results indicate that CoPP treatment tends to decrease the number of GFP+ bacteria per cell, while SnPP treatment increases the number of bacteria per cell. However, no significant differences are observed between the treatments. These results suggest that, at least at 4 h post-infection, CoPP does not use autophagy to eliminate *S*. Typhimurium in RAW264.7 cells.

## 4. Discussion

*Salmonella* infections are an important public health problem due to the capacity of this bacterium to cause persistent infections [44]. It is well described that Hmox1 has an immunomodulatory function in bacterial infections, both in vitro and in vivo [45]. Likewise, Hmox1 or its product CO has been proposed as a target for treatments for many diseases [46]. In this study, we explored the effects of Hmox1 induction or inhibition in the outcome of a model of acute and persistent infection caused by *S.* Typhimurium in mice.

In the initial phase of *S.* Typhimurium infection in mice, the bacterium reaches deep organs and blood, and induces death before day 10 post-infection [6]. CoPP treatment showed a tendency to reduce clinical score, allowing the survival of 20% of mice (Figure 1A,B). Surprisingly, similar results were observed in SnPP-treated mice. SnPP is frequently used as a control with an opposite effect to CoPP, as this drug acts by inhibiting the activity of Hmox1 through competition with its substrates; however, it may increase HO expression [21]. Because of this, we believed that the inhibition of HO activity by SnPP in this model of acute infection may result in the induction of *hmox1* gene expression and increase Hmox1 protein production, as we observed in the spleen of uninfected mice (Figure A2). This could lead to the activation of additional genes involved in host defense, which are not necessarily related to the enzymatic activity of Hmox1 and its products.

IL-10 is a crucial mediator of the immune response to *S.* Typhimurium [47] and previous data from our laboratory have described that IL-10 has a peak of expression at day 5 post-infection with *S.* Typhimurium present in the ileum, liver, and spleen of C57BL/6J mice [6]. As shown in Figure 1E, *S.* Typhimurium infection effectively induced IL-10 expression, but it was reduced in CoPP-treated mice, when compared with vehicle and SnPP-treated mice. This result could suggest that CoPP treatment limits the systemic spread of the bacteria, which results in less IL-10 production. However, because IL-10 is involved in the signaling pathway of *Hmox1* transcription [48,49], the phenotype observed may be due to negative feedback: if Hmox1 is highly produced, IL-10 could be suppressed. In both cases, a reduced amount of *S.* Typhimurium colonizing the tissues should be observed. Therefore, we decided to quantify the bacterial burden in mice treated with CoPP, SnPP, and vehicle. Bacterial loads from spleen, liver, mesenteric lymph nodes, gallbladder, feces, and blood were measured at 5 days post-infection. It is important to mention that CoPP is found in blood until day 3 after subcutaneous injection, but it is possible to detect cobalt in deeper organs after 4 weeks [50]. We observed that CoPP treatment could reduce bacterial loads when compared with vehicle-treated mice in the spleen, feces, and gallbladder, indicating an important effect of Hmox1 in bacterial clearance in acute *S.* Typhimurium infection. However, a different result was observed in the blood, where CoPP treatment significantly increased bacterial loads. This result may be explained by the higher iron availability in the blood caused by CoPP treatment that allows extracellular bacteria to replicate more in this case than under normal conditions [51]. To evaluate this hypothesis, we measured ferritin in serum by ELISA, as an indirect way of obtaining iron amounts in the blood. No significant differences were observed in ferritin amounts between the groups of mice (Figure A1), suggesting that higher availability of iron is not the reason why mice have higher bacterial loads in the blood, or at least not by itself. One of the main mechanisms to eliminate bacteria in the blood is through oxidation by erythrocytes [52], which is likely reduced in Hmox1 induction, preventing effective clearance. Moreover, the CO released by HO in the blood may be captured by hemoglobin to form carboxyhemoglobin, avoiding its bactericidal effect on the circulating bacteria. In addition, it must be considered that there may be additional mechanisms able to clear bacteria in the blood that could be HMOX-dependent or independently such as the production of innate molecules such as antimicrobial peptides and complement and/or the secretion of antibodies [53].

In the present work, during *S.* Typhimurium persistent infection, we observed that a constant induction of Hmox1 results in a reduced presence of bacteria in organs, as observed in Figure 3, but most importantly, when Hmox1 induction is initiated after *S.* Typhimurium infection begins and is already disseminated, bacteria were eliminated from all organs of CoPP-treated mice. It is worth mentioning that in the vehicle-treated group, not all mice have persistence, as our model includes antibiotic treatment, and that 24 h after intragastric infection, effective colonization of *S.* Typhimurium in mice was confirmed by plating feces in *Salmonella*-*Shigella* agar (data not shown). These results confirm that CoPP used as a treatment after *S.* Typhimurium infection avoided persistent infection several days after the inoculation.

All the data observed in vivo were confirmed in vitro. CoPP effectively reduced *S.* Typhimurium survival inside RAW264.7 cells, and SnPP was able to allow higher survival of the bacterium compared with vehicle-treated cells. To evaluate the mechanism behind this phenomenon, we treated these cells with CORM3. However, no significant differences were observed with none of the concentrations used.

In addition to the important effect of Hmox1 activity and its products in controlling inflammatory diseases and bacterial infections, porphyrins alone have also been studied as possible treatments for bacterial infections [54,55]. Although metalloporphyrins, such as CoPP and SnPP, have a very similar structure and can mimic some effects of heme, these have many differences in their antimicrobial effect. Bacteria that do not express heme uptake systems, such as *Salmonella* [56], can be resistant to metalloporphyrins. Such is the case of *E. coli*, which is resistant to gallium-protoporphyrin-IX [57]. However, we tested the effect of CoPP and SnPP in *S*. Typhimurium growth in vitro, and no differences were observed between treatments and vehicle or untreated (data not shown).

Previous studies have proposed autophagy as one of the mechanisms that control the survival of intracellular pathogens and could explain the decreased survival of *S*. Typhimurium in our in vitro assays. These studies proposed a key role for autophagy in restricting the replication of intracellular bacteria [35,58]. Intracellular *S*. Typhimurium is targeted by ubiquitination and subsequent recruitment of proteins required for autophagosome formation, including p62 and the ATG8 family member protein, LC3 [59]. A study conducted by Birmingham and collaborators determined that autophagy restricts bacterial growth, since cells with altered autophagy are more permissive to the growth of *S*. Typhimurium than wild-type cells [41]. However, it is known that *S*. Typhimurium can manipulate autophagy, suppressing it through the production of reactive persulfides, thus avoiding cell death [60,61]. On the other hand, previous studies have evaluated the effect of Hmox1 induction on LPS-induced autophagy in rat liver and RAW264.7 cells. Both studies evaluate autophagy markers as LC3 and propose that CoPP treatment induces autophagy in these models [62,63]. Unuma et al. propose that autophagy is induced with faster kinetics in rats treated with LPS and CoPP, since after 1 h of treatment significant differences were observed in the activation of LC3 as well as in the degradation of p62. This could be related to and explain our results in which no differences are seen in the level of co-localization of *S*. Typhimurium with LC3 and LAMP-1 at 4 h post-infection (Figure 6 and Figure 7), since autophagy was likely induced before that evaluation [41,42].

The role of Hmox1 in metabolism has been well studied, and it was recently described that this enzyme is crucial to the correct function of mitochondria in lung epithelial cells. Using a Hmox1 knockout lung cell line, it was observed that Hmox1 contributes to electron transport chain activity and its absence results in lower oxygen consumption rates [64]. Hmox1 can also induce mitochondrial biogenesis and reduce lethal *Staphylococcus aureus* sepsis [65] and specifically in macrophages, Hmox1 can stimulate phagocytosis [66]. Given this, it would be important to explore mitochondrial function in our in vitro model to evaluate if this pathway is involved in *S.* Typhimurium elimination.

Although the use of metalloporphyrins such as CoPP and SnPP to modulate HO activity has been previously described [19,22], there are certain limitations in our model of persistent infection that do not allow us to ensure an increase or inhibition in HO activity with the use of these metalloporphyrins. Limitations such as sample size, assays performed, or repetitions of these assays could be incorporated in future studies. On the other hand, it is worth mentioning that the persistent *S.* Typhimurium infection model used in this work was established in our laboratory to evaluate whether prior *S.* Typhimurium infection plays a role in the development of inflammatory bowel disease (IBD) in an IL-10^−/−^ mouse model. To corroborate that the inflammation observed in the intestinal epithelium was due to the onset of IBD and not to the presence of the bacteria in the intestine, we treated the mice with the antibiotic Enrofloxacin after infection. Surprisingly, most of the mice still had the bacteria despite antibiotic treatment [9]. However, this persistent model in wild-type mice showed a variable rate of *Salmonella* persistence, as observed in this study. Moreover, SnPP and ZnPP have been reported to inhibit not only Hmox1, but also HO-2 [67,68], which may result in a general inhibition of HO activity in vivo, and further studies are required to elucidate the contribution of each isoform in the effects described in this work.

The potential of Hmox1 and its derivatives as a treatment or target to treat inflammatory and infectious diseases has been widely discussed [69,70,71], and the data presented herein highlight this potential. However, further work is required to elucidate the exact mechanism by which Hmox1 would be exerting this effect in the *S.* Typhimurium persistence model. We anticipate that, given the evidence that persistent *S.* Typhimurium resides inside macrophages for long periods [10,11], bacteria may be modulating Hmox1 expression or activity, allowing its survival, but not its upgrowth, thus avoiding antigen presentation and immune system activation.

## Figures and Tables

**Table 1:** *S.* Typhimurium load in mice organs after prophylactic treatment with CoPP.

		*Salmonella* Typhimurium Load ^1^			
Experimental Group	Mouse	Liver (CFU/mg)	Spleen (CFU/mg)	mLN (CFU/mg)	Blood (CFU/mL)
STm pre-Veh	1	>5000	1.44	-	-
	2	-	-	-	-
	3	>5000	>5000	4.4	333.3
	4	-	-	-	-
	5	-	-	-	-
	6	-	-	-	-
	7	-	-	-	-
	8	-	-	-	-
STm pre-CoPP	1	41.7			
	2	-	-	-	-
	3	-	-	-	-
	4	-	-	-	-
	5	-	-	-	-
	6	-	-	-	-
	7	-	-	-	-
	8	-	-	-	-
	9	-	-	-	-
	10	-	-	-	-
STm pre-SnPP	1	181.8	-	-	-
	2	-	-	-	-
	3	-	-	-	-
	4	-	>5000	-	-
	5	-	-	-	-
	6	-	>5000	-	466.7
	7	-	-	-	-
	8	-	-	-	-
	9	-	-	-	-
	10	-	-	-	-
	11	-	-	-	-
	12	-	-	-	-
	13	-	-	-	-
	14	6.41	12.9	-	-

^1^ Female C57BL/6 mice were treated with CoPP, SnPP, or its vehicle 24 h before intragastric infection with 1 × 10^6^ CFU of *S.* Typhimurium. Bacterial loads were quantified at day 38 post-infection in blood spleen, liver, mesenteric lymph nodes (mLN), and feces. Bacterial loads of feces are not shown because no CFUs were observed.

**Table 2:** *S.* Typhimurium load in mice organs after post-infection treatment with CoPP.

		*Salmonella* Typhimurium Load ^1^			
Experimental Group	Mouse	Liver (CFU/mg)	Spleen (CFU/mg)	mLN (CFU/mg)	Blood (CFU/mL)
STm post-Veh	1	-	171.8	-	-
	2	-	61.7	-	-
	3	-	61.7	-	-
	4	-	-	-	-
	5	-	-	-	-
STm post-CoPP	1	-	-	-	-
	2	-	-	-	-
	3	-	-	-	-
	4	-	-	-	-
	5	-	-	-	-
	6	-	-	-	-
STm post-SnPP	1	-	13.6	-	-
	2	-	95.5	-	-
	3	-	-	-	-
	4	-	>5000	>5000	>5000
	5	-	11	40.9	-
	6	-	-	-	-
	7	-	-	-	-
	8	-	-	-	-
	9	-	-	-	-
	10	-	-	-	-
	11	-	-	-	-
	12	-	-	-	-

^1^ Female C57BL/6 mice were treated with CoPP, SnPP, or its vehicle 72 h after intragastric infection with 1 × 10^6^ CFU of *S.* Typhimurium. Bacterial loads were quantified on day 38 post-infection in blood spleen, liver, mLN, and feces. Bacterial loads of feces are not shown because no CFUs were observed.

## Data Availability

Data is contained within the article.

## Appendix A

**Table A1:** Daily supervision chart for *S.* Typhimurium infected C57BL/6J female mice.

	Observations	Score
Physiological parameters: -weight loss	Normal (no weight loss)	0
	Weight loss under 10%	1
	Weight loss between 10–20%	2
	Weight loss greater than 20%	3
Appearance parameters: -posture	Normal (erect, intense activity, clean fur)	0
	Lower activity, hirsute fur, presence, or absence of porphyric secretion in eyes and nose	1
	Bowed position, very low activity, stays at the bottom of the cage, hirsute fur, porphyric secretion in eyes and nose	2
	Prostate, lateral decubitus, obvious dehydration, hollow eyes	3
Spontaneous behavior: -response to stimuli	Normal (watchful, interacts with mates, groom)	0
	Little changes: less groom and motion, accelerated breathing, tends to stay at the bottom of the cage	1
	Wobbly motion, inactive or stays at the bottom of the cage, abdominal breathing	2
	Motionless, prostrate, lateral decubitus, open its mouth to breath	3

Total score: 12. Score from 0 to 3: normal *; from 4 to 6: supervise carefully; from 7 to 9: intense suffering. Consider and evaluate euthanasia **; from 10 to 12: practice euthanasia immediately. * When one mouse has reached score 3 in more than one parameter, all “3” must be considered as “4”. ** Score from 8 to 12 are indicators of significant deterioration and intense suffering. Therefore, euthanasia must be considered. If this happens, anesthetic overdose will be applied followed by cervical dislocation.
